# Diabetes duration and weight loss are associated with onset age and remote metastasis of pancreatic cancer in patients with diabetes mellitus

**DOI:** 10.1111/1753-0407.13259

**Published:** 2022-02-15

**Authors:** Minglei Ma, Wei Li, Lingling Xu, Fan Ping, Huabing Zhang, Yuxiu Li

**Affiliations:** ^1^ Department of Endocrinology, Key Laboratory of Endocrinology, Ministry of Health Peking Union Medical College Hospital, Peking Union Medical College, Chinese Academy of Medical Sciences Beijing China; ^2^ Department of Endocrinology, Beijing Shijitan Hospital Capital Medical University Beijing China

**Keywords:** anti‐diabetic drugs, diabetes mellitus, pancreatic cancer, weight loss, 胰腺癌, 糖尿病, 减肥, 降糖药物

## Abstract

**Objective:**

To analyze the clinical characteristics of patients with pancreatic cancer (PC) and diabetes and to explore the impact of diabetes duration, weight loss, and hypoglycemic drugs on the tumor biological behavior of PC.

**Methods:**

This is a retrospective study on patients with PC and diabetes. Subjects were grouped according to the onset age of PC, distant metastasis, duration of diabetes, degree of weight loss (∆Wt), and type of hypoglycemic drugs. Logistic regression analysis was used to evaluate the association between diabetes duration, weight loss, hypoglycemic drugs, and early‐onset PC, distant metastasis.

**Results:**

Compared with late‐onset PC, patients with early‐onset PC had a higher proportion of new‐onset DM (35 [79.5%] vs. 217 [46.9%], *p* < 0.001), smoker, drinker, and more obvious weight loss (8.5 [3.8, 15] kg vs. 5 [0, 10] kg, *p* < 0.001). Patients with remote metastasis had an earlier diagnosis age, heavier weight loss, lower body mass index, and were more likely to be smokers but had cancer less likely to be localized in the head of pancreas. Regression analysis showed that new‐onset diabetes and weight loss were independently correlated to early‐onset PC: odds ratio (OR) = 3.38 (95% CI 1.36‐8.4, *p* = 0.09; OR = 1.56 (95% CI 1.16‐2.1), *p* = 0.003, respectively. In contrast, long‐term diabetes, and heavy weight loss were independently associated with remote metastasis: OR = 3.38 (95% CI 1.36‐8.4, *p* = 0.09; OR = 1.56 (95% CI 1.16‐2.1), *p* = 0.003, respectively.

**Conclusion:**

New‐onset diabetes and weight loss were common presentation and risk factors of early‐onset PC, which required more attention. Long‐term diabetes and heavy weight loss were risk factors contributing to distant metastases, indicating potential risk factors contributing to the adverse prognosis of patients with PC.

## Graphical abstract

**Highlights**

- New‐onset diabetes and weight losses were common presentation and risk factors of early‐onset PC, which required more attention.
- Long‐term diabetes and heavy weight loss were risk factors contributing to distant metastases and less radical surgery, which needed to be better treated.

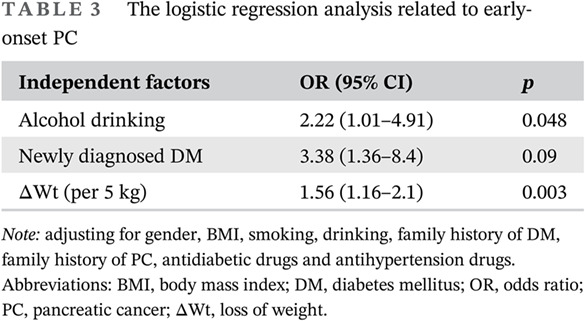

## 摘要

**目的:**

分析胰腺癌(PC)合并糖尿病患者的临床特点, 探讨糖尿病病程、体重减轻及降糖药物对PC肿瘤生物学行为的影响。

**方法:**

对PC合并糖尿病患者进行回顾性研究。受试者根据PC的发病年龄、远处转移、糖尿病病程、体重减轻程度(∆Wt)和降糖药物的类型进行分组。采用Logistic回归分析评估糖尿病病程、体重减轻、降糖药物与早发性PC、远处转移之间的关系。

**结果:**

与晚发PC相比, 早发PC患者新发DM比例更高(35[79.5%]vs 217[46.9%], p<0.001), 吸烟、饮酒和体重减轻更明显(8.5[3.8, 15]kg vs 5[0, 10]kg, p<0.001)。远处转移的患者诊断年龄较早, 体重减轻较大, 体重指数较低, 且更有可能是吸烟者, 但癌症不太可能发生在胰头。回归分析显示, 新发糖尿病和体重减轻与早发PC独立相关:分别为优势比(OR)=3.38(95%置信区间(CI) 1.36~8.4, P=0.09), OR=1.56(95%CI 1.16~2.1), P=0.003。相反, 长期糖尿病和严重的体重减轻与远处转移独立相关:分别为OR=3.38(95%CI 1.36‐8.4, p=0.09, OR=1.56(95%CI 1.16‐2.1), p=0.003)。

**结论:**

新发糖尿病和体重减轻是早发性PC的常见表现和危险因素, 应引起更多重视。长期糖尿病和体重减轻是导致远处转移的危险因素, 这表明潜在的危险因素导致了PC患者的不良预后。

## 1 INTRODUCTION

Many studies have shown that diabetes mellitus (DM) is related to an increased risk of various tumors.^1^, ^2^ Among these tumors, pancreatic cancer (PC) is closely related to DM. The causal relationship between PC and DM can be bidirectional, and the causality is closely related to the duration of DM. Studies have identified DM as a relatively definite risk factor for PC. A pooled analysis found that DM with a duration of more than two years significantly increased the risk of PC (odds ratio [OR] = 1.9, 95% confidence interval [CI] 1.72–2.09), even when the course of DM extended to 20 years, the risk of PC was still significantly increased (OR = 1.3，95% CI 1.03–1.63).^3^ The prevalence of PC in newly diagnosed diabetic patients within three years is up to 0.85%, which is 6‐8 times that of the general population.^4^ The risk of PC in newly diagnosed DM (hazard ratio [HR] = 3.71, 95% CI 2.83–4.88) is 2.3 times that of long‐term diabetes (HR = 1.61, 95% CI 1.18–2.21).^5^ Besides, up to 85% of patients with PC were associated with DM or impaired glucose tolerance at diagnosis. The proportion of newly diagnosed DM in patients with PC is up to 52%–74%.^6^, ^7^ Therefore, it is currently believed that new‐onset DM is not only a risk factor of PC but also a possible early manifestation of PC,^8^ thus facilitating the early detection of PC. Therefore, understanding the clinical characteristics of patients with PC and DM can help better understand the relationship between the two diseases and promote the screening, early recognition, and early diagnosis of PC.

Weight loss is one of the clinical manifestations of many tumors, and severe weight loss can be manifested as cachexia. The degree of weight loss often reflects the rate of progression and degree of tumor’s malignancy. PC is highly malignant and is the fourth leading cause of tumor death in the United States, with a 5‐year survival rate less than 9%.^9^ However, because of the occult onset and nonspecific presentation, more than half of patients with PC were diagnosed at an advanced stage with remote metastasis,^9^ thus losing the chance to operate radical surgery, the only possible curable treatment of PC.^10^ Therefore, a better understanding of the clinical manifestation and risk factors of PC will help identify high‐risk populations early and achieve early diagnosis and proper treatment, thereby improving the survival rate. Studies have found that newly diagnosed type 2 diabetes mellitus (T2DM) is often accompanied by weight gain. In contrast, PC associated newly diagnosed DM is often accompanied by weight loss before the diagnosis of PC.^11^, ^12^ Therefore, weight loss is of great value in identifying the potential PC and the early diagnosis of PC in patients with newly diagnosed DM.^13^

In addition to the close relationship between DM and PC, many studies have found that antidiabetic drugs also affect the risk and survival of PC. Studies have shown that taking metformin reduced the risk of PC while taking sulfonylurea or insulin and its analogs significantly increased the risk of PC.^14^, ^15^, ^16^, ^17^ Cross‐sectional studies have shown that patients with PC and DM taking metformin had better overall survival compared with those without taking metformin.^18^, ^19^ However, two randomized controlled trials (RCT) have not found that metformin combined with chemotherapeutic drugs is beneficial to the survival of patients with advanced PC.^20^, ^21^ This negative impact of metformin may be due to the higher drug concentration required for metformin’s antitumor effect. Besides, the patients included were all at an advanced stage with remote metastases in these two RCT. Anyway, antidiabetic drugs might have certain impacts on the occurrence and prognosis of PC, and more attention is needed for the effects of antidiabetic drugs on the development of tumors in patients with PC and DM.

In this study, we collected data of patients diagnosed with PC and DM in Peking Union Medical College Hospital in the past 35 years to analyze the clinical characteristics and tumor biological behavior of these patients. We grouped the populations according to the onset age of PC, with or without remote metastasis, duration of DM, the degree of weight loss, and the type of antidiabetic drugs. We tried to explore the impact of different risk factors on PC prognosis from the perspective of effects on tumor biological behavior.

## 2 METHODS

### 2.1 Study population

**FIGURE 1:**
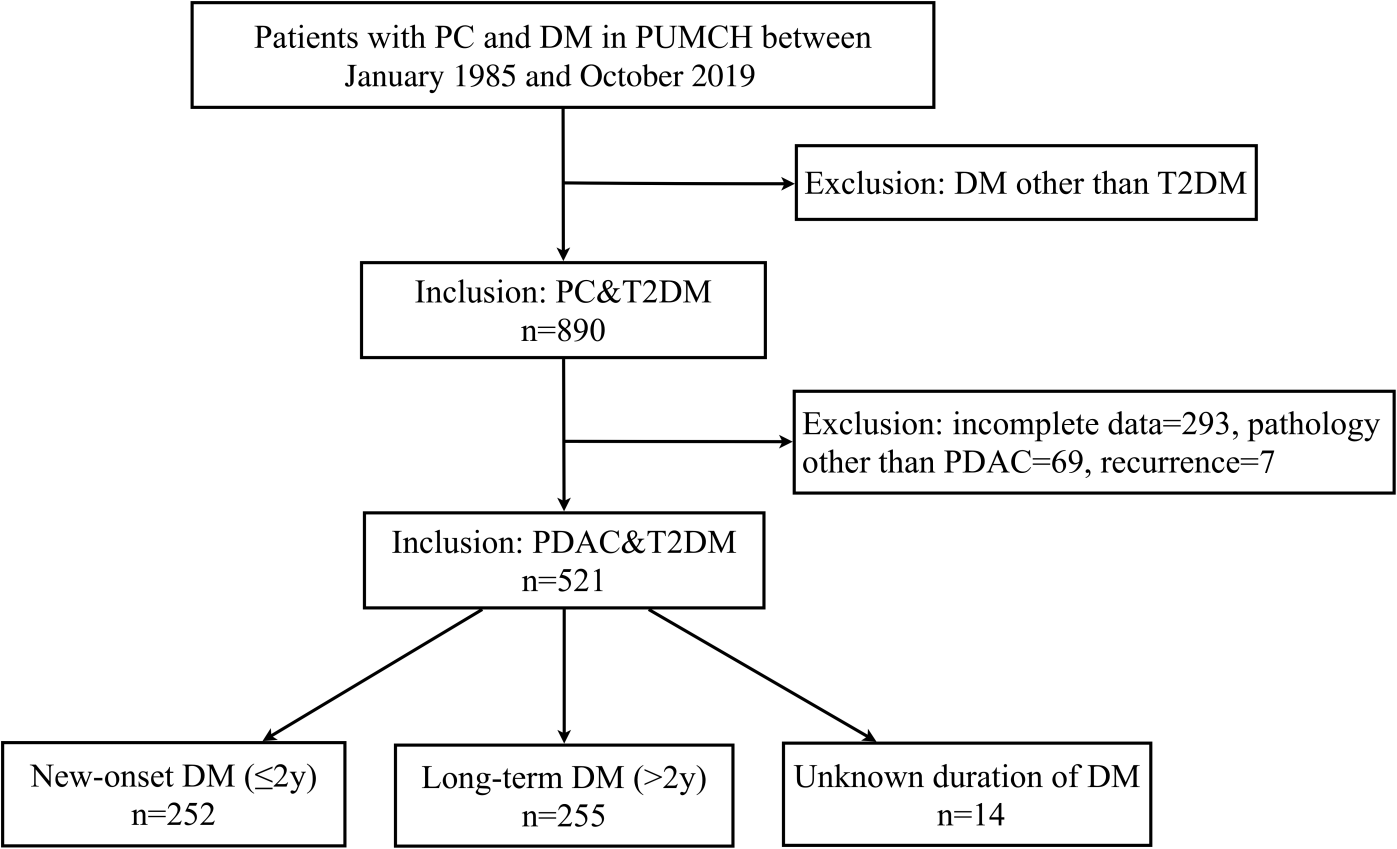
Study flow chart. DM, diabetes mellitus; PC, pancreatic cancer; PDAC, pancreatic ductal adrenal carcinoma; PUMCH, Peking Union Medical College Hospital; T2DM, type 2 diabetes mellitus

A retrospective study was conducted in patients with PC and DM hospitalized in Peking Union Medical College Hospital from January 1985 to October 2019. After excluding patients with type 1 diabetes (T1DM), gestational diabetes (GDM), and other special types of DM, a total of 890 cases of PC with T2DM were retrieved. Data were collected by reviewing their medical records. Then patients were excluded who had incomplete data (*n* = 293), tumor recurrence(*n* = 7), or diagnosed with pancreatic tumor other than pancreatic ductal adrenal carcinoma (PDAC, *n* = 69), including intraductal papillary‐mucinous neoplasms (*n* = 25), pancreatic neuroendocrine tumors (PNET, *n* = 15), mucinous cystic neoplasms (*n* = 12), cystadenocarcinoma (*n* = 5), adenosquamous carcinoma (*n* = 4), mucinous adenocarcinoma (*n* = 3), pancreatic small cell carcinoma (*n* = 2), multiple endocrine neoplasia, type1 (*n* = 1), acinar cell carcinoma (*n* = 1), and pancreatic polypeptide tumor (*n* = 1). Finally, 521 cases with histopathological confirmed PDAC with T2DM were included in this study (Figure 1). The study was conducted in accordance with ethical standards and was approved by the Ethics Committee of Peking Union Medical College Hospital.

### 2.2 Data collection

Data were collected by searching electronic and paper medical records. Basic information including age, gender, height, body weight was obtained, and body mass index (BMI, kg/m^2^) was calculated by dividing body weight (kg) by the square of height (m). Weight loss was collected through medical records, and weight before PC onset was calculated by current weight plus weight loss. BMI before onset of PC was calculated by using a similar formula. Information about smoking, drinking, past medical histories like hypertension, dyslipidemia, chronic pancreatitis, and medication history, including antihypertensive drugs, lipid‐lowering drugs, and hypoglycemic drugs, as well as family histories like DM, PC, and other cancers, were collected. Glycosylated hemoglobin (HbA1c) and fasting blood glucose (FPG) was obtained to assess blood glucose control.

### 2.3 Tumor related information

Tumor related information included tumor size, metastasis, tumor/node/metastasis (TNM) staging, surgical methods, pathological grade, chemotherapy, and tumor markers like carbohydrate antigen 199 (CA 19‐9), carcinoembryonic antigen (CEA), and carbohydrate antigen 242 (CA 242). The tumor’s size was obtained through the enhanced computed tomography scan of the abdomen or observations in the operation records. Tumor staging was assessed according to the eighth edition staging criteria of the American Joint Committee on Cancer. The types of surgery were divided into radical surgery and palliative surgery. Radical surgery includes pancreaticoduodenectomy, distal pancreatectomy, and total pancreatectomy. Palliative surgery was performed to relieve obstructive or compression symptoms when patients were intolerant to radical surgery. The pathological classification was made according to the differentiation of tumor cells and was divided into three grades: well differentiated, moderately differentiated, and poorly differentiated.

### 2.4 Definition and grouping

Early‐onset PC was defined as the onset age of PC less than 50 years old. Because of the rapid progress and short medical history of PC, the onset age of PC was considered equal to the diagnosis age. Thus, patients were grouped into two groups according to the onset age of PC: early‐onset PC (onset age less than 50 years old) and late‐onset PC (onset age more than 50 years old). DM was defined as one of the following: FPG ≥ 7.0 mmol/L, oral glucose tolerance test 2 h plasma glucose ≥11.1 mmol/L, previously diagnosed DM, or antidiabetic treatment already prescribed. T1DM, GDM, and other types of DM were excluded. New‐onset DM was defined as diabetic duration within two years, and long‐standing DM was defined as a diabetic duration for more than two years. Patients were divided into two groups according to the median weight loss of the overall population (5 kg): heavy weight loss group (ΔWt ≥5 kg) and the control group with slight or no weight loss (ΔWt < 5 kg). The assessment of antidiabetic drugs includes four drugs: metformin, α‐glycosidase inhibitors (AGI), sulfonylureas, and insulin. Patients were categorized as user or nonuser group according to these four antidiabetic drugs. Other medications (including dipeptidyl‐peptidase‐4 inhibitors, glucagon‐like peptide‐1, and sodium‐glucose cotransporter‐2) were less commonly used by these patients and, therefore, were not included in the analyses.

### 2.5 Statistical analysis

Normality was assessed for all continuous variables. Normally distributed data were expressed as the mean ± SD, and Student’s *t* test was used to assess differences between groups. Nonnormally distributed data were expressed as the median (Q1, Q3), and differences between groups were assessed using the Mann‐Whitney *U* test. Categorical variables were expressed as counts/percentiles (%), and Pearson’s χ2‐test was used to compare the difference between groups. Multivariable logistic regression analysis with forward variable selection procedure was conducted to explore the effect of variable factors, including DM duration, weight loss, and antidiabetic drugs on early‐onset PC, remote metastasis, and radical surgery. Confounding factors like age, gender, BMI, smoking, alcohol, FPG, hypertension, dyslipidemia, and family history of PC and DM were adjusted. All statistical analyses were performed using IBM SPSS Statistics 23.0 (IBM Corp., Armonk, NY, USA). A *p* value <0.05 was considered significant.

## 3 RESULTS

### 3.1 Basic characteristics of patients grouped by onset age of PC

The average age of patients with PC and DM was 62.9 years old, whereas the average age of early‐onset PC patients was 46.2 years old, far younger than the total and late‐onset PC patients. More than three quarters of early‐onset PC patients were male and newly diagnosed with DM, with a median duration of DM 8 months. More weight loss was observed in early‐onset PC patients than late‐onset PC patients (8.5 [3.8, 15] kg vs. 5 [0, 10] kg, *p* < 0.001), though the BMI was similar between the two groups. More patients with early‐onset PC were smoker and drinker and were more likely to had family history of PC and DM than those with late‐onset PC. Compared with late‐onset PC patients, those with early onset age suffered larger tumor size (4 [3, 6] cm vs. 3.5 [2.6, 4.4] cm, *p* = 0.013). A higher proportion of PC patients with early‐onset age received chemotherapy, whereas a lower proportion of those patients underwent radical surgery, though with no statistical significance (Table 1).

**TABLE 1:** Basic characteristic, tumor related characteristics and drugs between early‐onset PC and late‐onset PC

	Total	Early onset of PC (onset age ≤ 50 y)	Late onset of PC (onset age > 50 y)	
	*n* = 521	*n* = 44 (8.4%)	*n* = 477 (91.6%)	*p*
Age (yo)	62.9 ± 9	46.2 ± 4.3	64.4 ± 7.7	<0.001
Male	308 (59.1%)	34 (77.3%)	274 (42.6%)	0.01
Duration of DM (m)	36 (3, 120)	8 (1.3, 22.5)	36 (3, 120)	0.002
New‐onset DM (≤2y)	252 (49.7%)	35 (79.5%)	217 (46.9%)	<0.001
BMI (kg/m^2^)	23.5 ± 3	23.7 ± 3.8	23.4 ± 2.9	0.657
ΔWt (kg)	5 (0, 10)	8.5 (3.8, 15)	5 (0, 10)	0.001
Heavy weight loss (≥5 kg)	274 (58.3%)	31 (73.8%)	243 (56.8%)	0.033
Smoking	192 (39.3%)	24 (60%)	168 (37.4%)	0.005
Alcohol drinking	151 (31%)	23 (56.1%)	128 (28.7%)	<0.001
Family history of DM	84 (17.3%)	12 (28.6%)	72 (16.2%)	0.043
Family history of PC	10 (2%)	4 (9.5%)	6 (1.3%)	<0.001
Diameter of tumor (cm)	3.6 (2.6, 4.5)	4 (3, 6)	3.5 (2.6, 4.4)	0.013
Position of tumor (head)	306 (59.9%)	26 (60.5%)	280 (59.8%)	0.935
Remote metastasis	91 (20.6%)	9 (23.7%)	82 (20.3%)	0.622
TNM III‐IV	98 (22.5%)	9 (24.3%)	89 (22.4%)	0.837
Pathology‐low grade	113 (37.5%)	7 (35%)	106 (37.7%)	0.808
Radical surgery	280 (55.7%)	17 (43.6%)	263 (56.7%)	0.114
Chemotherapy	236 (71.3%)	20 (90.9%)	216 (69.9%)	0.035
CA 199 (U/ml)	232.9 (62.2, 623.6)	242.8 (96.2, 966.5)	226.2 (58.3, 606.3)	0.17
CEA (ng/ml)	4.3 (2.6, 8.5)	3.3 (1.6, 14.5)	4.3 (2.7, 8.4)	0.349
CA 242 (U/ml)	55.0 (12.6, 149.2)	62.5 (25.8, 150.0)	54.9 (12.0, 148.3)	0.365
FPG (mmol/L)	8.9 ± 3	8.5 ± 3	9 ± 3	0.364
HbA1c (%)	7.9 ± 1.6	8.1 ± 2.1	7.9 ± 1.6	0.624
Metformin	107 (24.6%)	14 (41.2%)	93 (23.2%)	0.019
AGI	89 (20.4%)	4 (11.8%)	85 (21.1%)	0.193
Sulfonylureas	96 (22.1%)	8 (23.5%)	88 (22%)	0.837
Insulin	215 (46.6%)	13 (37.1%)	202 (47.4%)	0.241

Abbreviations: AGI, alpha‐glucosidase inhibitor; BMI, body mass index; DM, diabetes mellitus; CA 19–9, carbohydrate antigen 199; CEA, carcinoembryonic antigen; CA 242, carbohydrate antigen 242; FPG, fasting plasma glucose; HbA1c, glycosylated hemoglobin; PC, pancreatic cancer; TNM, tumor/node/metastasis; ΔWt, loss of weight.

### 3.2 Characteristics among patients with or without remote metastasis

PC patients with remote metastasis were younger than those without metastasis; however, the proportion of early‐onset PC was similar between the two groups. Metastatic PC patients suffered more weight loss than those with localized tumor. Half of the patients with remote metastasis were smokers, and a higher proportion of them had family history of DM than those without remote metastasis. Less than half of the tumors in patients with remote metastasis were located in the head, a significant lower proportion compared to nonmetastatic patients (38 [43.2%] vs. 223 [63.7%], *p* < 0.001). The level of CA 19‐9, CEA, and CA 242 was higher in the metastatic group than the localized group (Table 2).

**TABLE 2:** The basic characteristics, tumorrelated characteristics, and drugs between PC patients with and without remote metastases

	Metastastic PC	Localized PC	
	*n* = 91 (20.6%)	*n* = 351 (79.4%)	*p*
Age (yo)	60.7 ± 9.5	63.1 ± 8.8	0.026
Male	65 (71.4%)	205 (58.4%)	0.023
Duration of DM (m)	36 (4, 120)	36 (3, 120)	0.49
Long‐term DM (>2y)	46 (52.3%)	168 (49.0%)	0.582
Early onset PC	9 (9.9%)	29 (8.3%)	0.622
BMI (kg/m^2^)	22.4 ± 2.6	23.8 ± 3.0	<0.001
ΔWt (kg)	6 (3, 10)	5 (0, 10)	0.02
Heavy weight loss (≥5 kg)	55 (69.6%)	178 (54.6%)	0.015
Smoking	40 (50.6%)	127 (37.8%)	0.036
Alcohol drinking	29 (36.3%)	107 (32%)	0.471
Family history of DM	21 (27.6%)	52 (15.5%)	0.012
Family history of PC	1 (1.1%)	4 (1.2%)	0.981
Diameter of tumor (cm)	4 (2.8, 4.7)	3.5 (2.6, 4.5)	0.082
Position of tumor (head)	38 (43.2%)	223 (63.7%)	<0.001
TNM III‐IV	90 (98.9%)	8 (2.3%)	<0.001
Pathology‐Low	14 (42.4%)	92 (36.9%)	0.569
Radical surgery	21 (24.7%)	241 (70.1%)	<0.001
Chemotherapy	52 (81.3%)	164 (68.6%)	0.047
CA 199 (U/ml)	324 (50.4, 2245.2)	215.75 (58.2, 520.8)	0.02
CEA (ng/ml)	6.6 (3.4, 16.1)	4.0 (2.5, 6.5)	<0.001
CA 242 (U/ml)	99.0 (19.6, 150.0)	47.7 (12.7, 120.6)	0.008
FPG (mmol/L)	9.0 ± 3.0	8.9 ± 2.9	0.851
HbA1c (%)	7.6 ± 1.4	8.0 ± 1.7	0.181
Metformin	16 (22.5%)	77 (25.1%)	0.653
AGI	23 (31.9%)	52 (16.9%)	0.004
Sulfonylureas	16 (22.9%)	65 (21.2%)	0.757
INS	39 (52.0%)	149 (46.1%)	0.359

Abbreviations: AGI, alpha‐glucosidase inhibitor; BMI, body mass index; CA 19–9, carbohydrate antigen 199; CEA, carcinoembryonic antigen; CA 242, carbohydrate antigen 242; DM, diabetes mellitus; FPG, fasting plasma glucose; HbA1c, glycosylated hemoglobin; PC, pancreatic cancer; TNM, tumor/node/metastasis; ΔWt, loss of weight.

### 3.3 Basic characteristics of patients grouped by duration of DM

Compared with patients with long‐term DM, PC was diagnosed earlier, and the proportion of early‐onset PC was higher in new‐onset DM. More weight loss was observed in new‐onset diabetic patients than long‐term diabetic patients, though the BMI was similar between the two groups. More patients with new‐onset DM had family history of PC, but fewer patients had family history of DM comparing to patients with long‐term DM. There was no significant difference in HbA1c levels between the two groups, whereas FPG levels were higher in the long‐term DM group (9.3 ± 3.1 mmol/L vs. 8.6 ± 2.9 mmol/L, *p* = 0.012). The proportions of antidiabetic drugs, including metformin, AGI, sulfonylureas, and insulin, were significantly higher in the long‐term DM group than in the new‐onset DM group. Patients with long‐term DM were more likely to have hypertension and dyslipidemia and take medications like antihypertensive drugs and lipid‐lowering drugs than the new‐onset DM group (Suppl. Table 1).

### 3.4 Characteristics among patients with different degrees of weight loss

The median weight loss in the heavy weight loss group was 10 (5, 10) kg, whereas the median weight loss in the control group was 0 (0, 3) kg. Compared with the control group, the heavy weight loss group had lower BMI, higher proportion of smoker and drinker, earlier diagnosed age of PC, higher proportion of early‐onset PC, shorter duration of DM, and higher proportion of new‐onset DM. Concerning the use of hypoglycemic drugs, metformin was more common in the heavy weight loss group than in the control group (68 [29.8%] vs. 29 [17.4%], *p* = 0.004). In terms of tumor related characteristics, compared with the control group, more patients in the heavy weight loss group had distant metastasis and advanced TNM stage, and fewer patients underwent radical surgery (119 [45.2%] vs. 138 [71.9%], *p* < 0.001) (Suppl. Table 2).

### 3.5 Comparison of tumor characteristics among people taking different hypoglycemic drugs

Compared with patients who did not take metformin, patients taking metformin suffered a more significant weight loss. However, the body weight and BMI before the onset of PC were higher than that of the control group. The proportion of early‐onset PC and nonmetastatic cases, was higher, and the tumor was smaller in those taking metformin than those not taking metformin. However, the ratio of radical surgery and grade of pathology was similar between the two groups. Compared with patients who did not take AGI, fewer patients taking AGI were in the early stage and more of them had distant metastasis and receiving chemotherapy. The ratio of radical surgery was lower in patients taking AGI than the controls, though with only a marginally significant difference. Compared with patients who did not take sulfonylureas, patients taking sulfonylureas were less likely to have radical surgery. There were no significant differences in patients taking insulin or not (Suppl. Table 3).

### 3.6 Logistic regression analysis to evaluate factors related to tumor biological behavior

Logistic regression analysis showed that, after adjusting for other confounding factors, alcohol consumption, new‐onset DM, and weight loss were independent risk factors for early‐onset PC (OR = 2.22, 95% CI 1.01‐4.91; OR = 3.38, 95% CI 1.36‐8.4; and OR = 1.56, 95% CI 1.16‐2.1 respectively) (Table. 3). Long‐term DM and heavy weight loss were not only independently correlated with distant metastasis (OR = 3.42, 95% CI 1.53‐7.67; and OR = 2.21, 95% CI 1.02‐4.79 respectively) but also were independent risk factors for reduced chance of radical surgery (OR = 0.46, 95% CI 0.22‐0.99; and OR = 0.39, 95% CI 0.18‐0.78 respectively) (Table 4, Suppl. Table 4).

**TABLE 3:** The logistic regression analysis related to early‐onset PC

Independent factors	OR (95% CI)	*p*
Alcohol drinking	2.22 (1.01–4.91)	0.048
Newly diagnosed DM	3.38 (1.36–8.4)	0.09
ΔWt (per 5 kg)	1.56 (1.16–2.1)	0.003

*Note:* adjusting for gender, BMI, smoking, drinking, family history of DM, family history of PC, antidiabetic drugs and antihypertension drugs.

Abbreviations: BMI, body mass index; DM, diabetes mellitus; OR, odds ratio; PC, pancreatic cancer; ΔWt, loss of weight.

**TABLE 4:** The logistic regression analysis related to remote metastasis

Independent factors	OR (95% CI)	*p*
BMI	0.83 (0.73–0.94)	0.005
Smoking	2.01 (1.01–4.25)	0.046
Long‐term DM	3.42 (1.53–7.67)	0.003
Heavy weight loss (∆Wt≥5 kg)	2.21 (1.02–4.79)	0.044
Position of tumor (head)	0.26 (0.12–0.54)	<0.001

*Note:* adjusting for age, gender, BMI, smoking, drinking, family history of DM, family history of PC, antidiabetic drugs, antihypertension drugs, position of the tumor and CA 19‐9.

Abbreviations: BMI, body mass index; CA 19‐9: carbohydrate antigen 199; DM, diabetes mellitus; OR, odds ratio; PC, pancreatic cancer; ΔWt, loss of weight.

## 4 DISCUSSION

This study showed that early‐onset PC had shorter duration of DM, heavier weight loss, larger tumor size, and higher proportion of smoker and drinker comparing to late‐onset PC. PC patients with remote metastasis were younger, leaner, and showing more obvious weight loss compared with those with local tumor. Compared with long‐term DM, PC patients with new‐onset DM were diagnosed earlier, and more obvious weight loss and larger tumor size were observed. However, PC patients with long‐term DM had higher FPG, were more likely to be complicated with hypertension and dyslipidemia, and more of them were taking various drugs. Regression analysis showed that new‐onset DM and weight loss were independent risk factors for early‐onset PC, whereas long‐term DM and heavy weight loss were independent risk factors for distant metastasis and less radical surgery.

In this study, we found that new‐onset diabetic patients were diagnosed with PC earlier and suffered a more obvious weight loss than those with long‐term DM. Researchers had reported that patients with newly diagnosed T2DM had an earlier onset age and were usually accompanied by weight gain. In contrast, patients with PC and new‐onset DM had a later onset age and were often accompanied by weight loss.^12^ What is more, new‐onset diabetic patients with heavy weight loss (>10%) could have significantly increased risk of PC.^22^ This is consistent with our finding that more prominent weight loss was seen in PC patients with newly diagnosed DM than those with long‐term DM. In fact, newly DM and weight loss may occur within 36 months before the diagnosis of PC and thus could be used as early manifestations of PC.^23^ This study showed that patients with new‐onset DM had not only a more obvious weight loss but also a larger tumor, which may jointly contribute to the prominent manifestations, thus leading to the early onset of PC. Therefore, new‐onset DM and weight loss could be used as manifestations relatively easy to recognize, thus prompting early detection of PC.

The regression analysis showed that new‐onset DM and weight loss were independent risk factors for early‐onset PC. This is consistent with another study showing that the duration of DM is positively correlated with the onset age of PC.^24^ A cohort study including 1407 cases of PDAC, 190 cases of PNET, and 100 cases of other types of pancreatic malignancies showed that patients with early‐onset pancreatic malignancies had better overall survival than those with late‐onset tumors (HR = 0.82，95% CI 0.67–1.0).^25^ However, in another cohort study of 72 906 cases of PC, the TNM stage was more advanced, and a higher proportion of patients in early‐onset PC had received radical surgery, radiotherapy, and chemotherapy; nevertheless, the 5‐year survival rate was lower (6.1% vs. 8.6%, *p* = 0.003).^26^ It is suggested that the biological behavior of early‐onset PC was more aggressive, so it is necessary to identify and improve the early screening of people at high risk of early‐onset PC.

Some researchers have proposed a PC risk prediction model in patients with new‐onset DM, a high‐risk population of PC. The risk of PC can be calculated and predicted based on the patient’s age, BMI, change of BMI, smoking, medication, and other biochemical indicators to calculate the risk of PC.^27^ Other scholars had proposed a simpler and more convenient method to predict the risk of PC by using age, change of weight, and change of blood glucose.^13^ In addition, this study also showed that new‐onset DM was more likely to have a family history of PC but less likely to have a family history of DM. Therefore, for newly diagnosed diabetic patients with significant weight loss but no family history of DM, special attention should be given to the possibility of PC.

The regression analysis in this study showed that long‐term DM was an independent risk factor for distant metastasis and reduced chance of radical surgery, suggesting that long‐term DM may be a poor prognostic risk factor for patients with PC. This is consistent with the results of a study that included three prospective cohorts. In this study, the author found that long‐term DM reduced the overall survival of patients with PC, whereas new‐onset DM had no adverse effect on the overall survival.^28^ In our study, patients with long‐term DM had higher FPG, were older, and more likely to have hypertension and dyslipidemia. It can be inferred that long‐term diabetic patients were more elderly, had more comorbidities, took more medications, and had poorly controlled blood glucose. This might be an important reason for the worse prognosis of patients with PC and long‐term DM.

Weight loss is a common clinical manifestation of PC. The causes of weight loss include malnutrition caused by anorexia and pancreatic enzyme deficiency and cachexia caused by metabolic changes and inflammation.^29^ In this study, patients who suffered heavy weight loss were diagnosed with PC earlier, and more of them were new‐onset DM comparing to the control group. As mentioned previously, new‐onset DM with heavy weight loss is an important clinical clue before PC diagnosis. This study showed that the proportion of advanced PC with distant metastasis was higher in patients with obvious weight loss. In comparison, the ratio of radical surgery in these patients was lower. The logistic regression analysis showed that heavy weight loss was an independent risk factor for distant metastasis and reduced radical surgery. Therefore, weight loss is not only an initial clinical manifestation helpful to the early detection and diagnosis of PC but also a reflection of highly malignant biological behavior indicating poor survival and prognosis of PC. Heavy weight loss and cachexia usually reflect the rapid tissue catabolism and the loss of muscle tissue with or without loss of adipose tissue,^30^ thus reducing the patient’s tolerance to surgery and postoperative survival.^31^ Therefore, for patients with PC suffering heavy weight loss, nutritional intervention, or pancreatic enzyme supplement therapy may be considered to avoid further weight loss and increase the tolerance of radical operation and improve the prognosis of PC.^29^

Different hypoglycemic drugs have different effects on tumor biological behavior. Patients taking metformin were more likely to have a less malignant tumor, including smaller tumor size, earlier TNM stage, and lower proportion of distant metastasis. This is consistent with other studies showing that patients with PC taking metformin have better overall tumor survival than those not taking metformin.^19^, ^32^ However, metformin’s beneficial effect on the prognosis of patients with PC was mainly seen in those with resectable tumors at an early stage,^18^ but not in those with remote metastasis at the advanced stage.^20^, ^21^, ^33^, ^34^ In this study, more massive weight loss and a higher proportion of early‐onset PC were seen in patients taking metformin. Besides, we also found that the weight and BMI before PC diagnosis were significantly higher in patients taking metformin than those without taking metformin. Therefore, we speculated that clinicians were more likely to choose metformin for overweight or obese diabetic patients before the diagnosis of PC. The use of metformin may also cause weight loss, which may promote the awareness to detect the potential PC. However, metformin caused weight loss is generally 2–4 kg, and heavy weight loss more than 5 kg is rare.^35^ Therefore, when new‐onset diabetic patients who were taking metformin showed heavy weight loss, clinicians should not only consider the adverse effect of metformin but also pay attention to the potential possibility of PC. And proper screening should be considered in patients with a high risk of PC.

Limitations and disadvantages: this study is a cross‐sectional study, which can only infer the correlation but could not determine the causal relationship. Therefore, cohort studies are needed to explore the influence of DM duration and weight loss on onset age and tumor biological behavior with PC. Besides, all the patients included in this study were diagnosed with PC and DM, but patients without PC and DM were not included. In addition, because of the cross‐sectional study, we could not access the survival and prognosis of patients with PC directly but only to assess the impact of factors on the biological behavior of tumors like onset age, distant metastasis, and radical surgery rate to infer the effects on tumor prognosis indirectly.

In summary, new‐onset DM and heavy weight loss were both common manifestations and independent risk factors of early‐onset PC. Therefore, it is necessary to pay special attention to the potential possibility of PC in newly diagnosed DM with obvious weight loss, especially those with other risk factors of PC. Long‐term DM and heavy weight loss might increase the risk of distant metastasis, reduce the chance of radical surgery, and thus may have adverse effects on the prognosis of patients with PC.

## DISCLOSURE

The authors declare no conflict of interest.

## Supporting information


**Appendix S1.** Supplementary Information
