# Supplementary material for: Diabetes duration and weight loss are associated with onset age and remote metastasis of pancreatic cancer in patients with diabetes mellitus
Source: J Diabetes. 2022 Feb 15;14(4):261–70. doi: 10.1111/1753-0407.13259 (PMC9060030; doi:10.1111/1753-0407.13259)
Supplement: Supplementary file 1 — Appendix S1. Supplementary Information [file JDB-14-261-s001.docx]

Suppl. table 1 Basic characteristic of people grouped by the duration of DM

|  | Total  (n=521) | New-onset DM  n=252 (49.7%) | Long-term DM  n=255 (50.3%) | *p* |
| --- | --- | --- | --- | --- |
| Age (year old) | 62.9±9 | 61.2±9.2 | 64.4±8.6 | <0.001 |
| Male | 308 (59.1%) | 148 (58.7%) | 151 (59.2%) | 0.912 |
| Duration of DM (month) | 36 (3, 120) | 3 (1, 12) | 120 (60, 180) | <0.001 |
| Onset of DM (yo) | 57.2±10.1 | 60.7±9.2 | 53.8±9.8 | <0.001 |
| Early-onset PC (≤50 yo) | 44 (8.4%) | 35 (13.9%) | 9 (3.5%) | <0.001 |
| BMI (kg/m2) | 23.5±3 | 23.5±3.1 | 23.4±2.9 | 0.907 |
| ΔWt (kg) | 5 (0, 10) | 5 (2.8, 10) | 5 (0, 7) | <0.001 |
| Smoking | 192 (39.3%) | 91 (39.4%) | 96 (38.7%) | 0.878 |
| Alcohol drinking | 151 (31%) | 79 (34.3%) | 68 (27.5%) | 0.107 |
| Family history of DM | 84 (17.3%) | 27 (11.8%) | 56 (22.6%) | 0.002 |
| Family history of PC | 10 (2%) | 8 (3.3%) | 2 (0.8%) | 0.047 |
| Diameter of tumor (cm) | 3.6 (2.6, 4.5) | 4 (3, 5) | 3 (2.5, 4) | <0.001 |
| Location of tumor (head) | 306 (59.9%) | 137 (55.9%) | 162 (64%) | 0.068 |
| Remote metastasis | 91 (17.6%) | 42 (16.9%) | 46 (18.1%) | 0.833 |
| TNM stage- III/IV | 99 (22.7%) | 45 (20.9%) | 51 (24.3%) | 0.075 |
| Pathology-low grade | 113 (37.5%) | 51 (34.5%) | 60 (41.4%) | 0.232 |
| Radical surgery | 280 (55.7%) | 140 (57.6%) | 134 (54.3%) | 0.373 |
| Chemotheraphy | 236 (71.3%) | 117 (75.5%) | 111 (66.9%) | 0.089 |
| CA 19-9 (U‎/ml) | 232.9(62.15,623.6) | 209.53(44.93,513.72) | 241.4(92.33,729.75) | 0.052 |
| CEA (ng‎/ml) | 4.31(2.61,8.48) | 3.99(2.23,6.93) | 4.63(2.8,9.19) | 0.109 |
| CA 242 (U‎/ml) | 54.95(12.63,149.18) | 47.7(11,118.3) | 57.45(14.75,150) | 0.197 |
| FPG (mmol‎/L) | 8.9±3 | 8.6±2.9 | 9.3±3.1 | 0.012 |
| HbA1c (%) | 7.9±1.6 | 8±1.8 | 7.8±1.4 | 0.387 |
| Metformin | 107 (24.6%) | 40 (19.5%) | 65 (29.4%) | 0.018 |
| AGI | 89 (20.4%) | 33 (16.1%) | 56 (25.2%) | 0.02 |
| SUs | 96 (22.1%) | 36 (17.6%) | 58 (26.2%) | 0.033 |
| INS | 215 (46.6%) | 63 (30%) | 147 (61%) | <0.001 |
| Hypertension | 253 (51.7%) | 104 (44.6%) | 142 (58.4%) | 0.003 |
| Dyslipidemia | 46 (10.1%) | 15 (7%) | 30 (13%) | 0.034 |
| Anti-hypertension drugs | 199 (42.4%) | 78 (35.1%) | 118 (49.8%) | 0.002 |
| lipid-lowering drugs | 30 (6.5%) | 6 (2.8%) | 23 (9.9%) | 0.002 |

Note：DM: diabetes mellitus; PC: pancreatic cancer; BMI: body mass index; ΔWt: loss of weight; CA 19-9: carbohydrate antigen 199 ; CEA: carcinoembryonic antigen; CA 242: carbohydrate antigen 242; FPG: fasting plasma glucose; HbA1c: Glycosylated hemoglobin; AGI: alpha-glucosidase inhibitor; SUs: Sulfonylureas; INS: insulin.

Suppl. table 2 Clinical and tumor characteristics among patients with different degrees of weight loss

|  | Heavy Wt loss  （ΔWt≥5kg） | Control  （ΔWt<5kg） |  |
| --- | --- | --- | --- |
|  | n=274 (58.3%) | n=196 (41.7%) | *p* |
| Age (year old) | 61.5±9.4 | 64.4±7.9 | <0.001 |
| Male | 172 (62.8%) | 110 (56.1%) | 0.147 |
| BMI (kg/m2) | 22.9±2.9 | 24.2±3 | <0.001 |
| ΔWt (kg) | 10 (5, 10) | 0 (0, 3) | <0.001 |
| Duration of DM (month) | 12 (2, 84) | 54 (2.3, 141) | 0.005 |
| Onset age of DM (yo) | 56.8±10.1 | 57.1±10.1 | 0.767 |
| Early-onset PC (≤50 yo) | 31 (11.3%) | 11 (5.6%) | 0.033 |
| Smoking | 114 (44%) | 63 (33%) | 0.018 |
| Alcohol drinking | 91 (35.3%) | 49 (25.8%) | 0.032 |
| Diameter of tumor (cm) | 3.5 (2.6, 4.7) | 3.6 (2.6, 4.4) | 0.763 |
| Location of tumor (head) | 172 (64.2%) | 106 (54.4%) | 0.035 |
| Remote metastasis | 55 (20.1%) | 24 (12.4%) | <0.001 |
| Radical surgery | 119 (45.2%) | 138 (71.9%) | <0.001 |
| Chemotherapy | 113 (69.8%) | 98 (69%) | 0.889 |
| TNM stage-III/IV | 56 (24.3%) | 29 (17.0%) | 0.005 |
| Pathology-low grade | 51 (37.5%) | 55 (38.7%) | 0.806 |
| CA 19-9 (U‎/ml) | 241.75(74.77,683.58) | 189.4(47.35,529.10) | 0.057 |
| CEA (ng‎/ml) | 4.76(2.70,9.12) | 3.9(2.29,6.70) | 0.028 |
| CA 242 (U‎/ml) | 56.6(12.98,149.18) | 44.5(7.98,128.75) | 0.185 |
| FPG (mmol‎/L) | 8.9±3 | 9±3 | 0.736 |
| HbA1c (%) | 8±1.9 | 7.7±1.1 | 0.209 |
| Met | 68 (29.8%) | 29 (17.4%) | 0.004 |
| AGI | 48 (21%) | 31 (18.6%) | 0.555 |
| SUs | 54 (23.8%) | 32 (19.2%) | 0.272 |
| INS | 103 (42%) | 87 (50%) | 0.107 |

Note：DM: diabetes mellitus; PC: pancreatic cancer; BMI: body mass index; ΔWt: loss of weight; CA 19-9: carbohydrate antigen 199 ; CEA: carcinoembryonic antigen; CA 242: carbohydrate antigen 242; FPG: fasting plasma glucose; HbA1c: Glycosylated hemoglobin; Met: metformin; AGI: alpha-glucosidase inhibitor; SUs: Sulfonylureas; INS: insulin.

Suppl. table 3 Comparison of tumor characteristics among patients taking different hypoglycemic drugs

|  |  | Metformin | | | AGI | | | SUs | | | Insulin | |
| --- | --- | --- | --- | --- | --- | --- | --- | --- | --- | --- | --- | --- |
|  | | Yes, n=107 | No, n=328 | Yes, n=89 | | NO, n=347 | Yes, n=96 | | No, n=340 | Yes, n=215 | | No, n=246 |
| Age (year old) | | 62±9.2 ^*^ | 63.6±9.1 | 64.8±8.7 | | 62.8±9.2 | 64.9±10 ^*^ | | 62.7±8.8 | 62.8±8.3 | | 63.4±9.5 |
| Male | | 58 (54.2%) | 194 (59.1%) | 50 (56.2%) | | 202 (58.2%) | 57 (59.4%) | | 195 (57.7%) | 134 (62.3%) | | 135 (54.9%) |
| BMI (kg/m^2^) | | 24.1±3.1^*^ | 23.3±3 | 23.2±3.4 | | 23.6±2.9 | 23±2.9 | | 23.7±3.1 | 23.6±2.9 | | 23.4±3.1 |
| ΔWt (kg) | | 5 (3, 10) ^*^ | 5 (0, 10) | 5 (2, 8) | | 5 (0, 10) | 5 (0, 10) | | 5 (0, 10) | 5 (0, 10) | | 5 (0.5, 10) |
| BMI before diagnosis (kg/m^2^) | | 26.3±3.3 ^**^ | 25.1±3.1 | 25.2±3.6 | | 25.5±3 | 25±3 | | 25.5±3.2 | 25.4±2.9 | | 25.5±3.3 |
| Wt before diagnosis (kg) | | 74.6±12.7 ^**^ | 70.3±11.8 | 69.3±13.4 | | 71.9±11.8 | 71.8±12 | | 71.3±12.2 | 72.8±11.4 | | 70.5±12.8 |
| New-onset DM | | 40 (38.1%) ^*^ | 165 (51.4%) | 33 (37.1%) ^*^ | | 172 (50.9%) | 36 (38.3%) ^*^ | | 168 (50.8%) | 63 (30%) ^***^ | | 147 (61%) |
| Early-onset PC | | 14 (13.1%) ^*^ | 20 (6.1%) | 4 (4.5%) | | 30 (8.6%) | 8 (8.3%) | | 26 (7.7%) | 13 (6%) | | 22 (8.9%) |
| Diameter of tumor (cm) | | 3 (2.4,4.3) ^*^ | 3.7 (2.8,4.7) | 3.6 (2.6,4.8) | | 3.5 (2.7,4.5) | 3.6 (2.8,5) | | 3.6 (2.6,4.5) | 3.4 (2.6,4.5) | | 3.6 (2.7,4.5) |
| Position of tumor (head) | | 69 (65.7%) | 191 (59.5%) | 49 (56.3%) | | 211 (62.1%) | 59 (64.1%) | | 200 (60.1%) | 127 (60.2%) | | 151 (62.7%) |
| Radical surgery | | 64 (61%) | 187 (58.1%) | 43 (49.4%) | | 208 (61%) | 44 (47.3%) ^*^ | | 207 (62.2%) | 118 (57%) | | 146 (60.3%) |
| Chemotherapy | | 45 (60.8%) | 160 (72.7%) | 53 (85.5%) ^**^ | | 153 (65.7%) | 39 (72.2%) | | 165 (69%) | 101 (69.7%) | | 112 (70.4%) |
| Stage of TNM | |  |  |  | |  |  | |  |  | |  |
| Stage I | | 44 (48.4%) ^*^ | 93 (33.2%) | 20 (27.4%) ^*^ | | 117 (39.1%) | 29 (36.3%) | | 108 (37.2%) | 67 (36.2%) | | 78 (37.9%) |
| Stage II | | 32 (35.2%) | 125 (44.6%) | 29 (39.7%) | | 128 (42.8%) | 33 (41.3%) | | 124 (42.8%) | 74 (40%) | | 90 (43.7%) |
| Stage III | | 0 (0%) | 7 (2.5%) | 1 (1.4%) | | 6 (2%) | 2 (2.5%) | | 5 (1.7%) | 6 (3.2%) | | 2 (1%) |
| Stage IV | | 15 (16.5%) | 55 (19.6%) | 23 (31.5%) ^*^ | | 48 (16.1%) | 16 (20%) | | 53 (18.3%) | 38 (20.5%) | | 36 (17.5%) |
| Metastasis | |  |  |  | |  |  | |  |  | |  |
| Remote | | 16 (15%) | 55 (17%) | 23 (26.1%) ^*^ | | 49 (14.2%) | 16 (16.7%) | | 54 (16.2%) | 39 (18.2%) | | 36 (14.8%) |
| Lymph node | | 15 (14%) | 79 (24.4%) | 16 (18.2%) | | 78 (22.7%) | 18 (18.8%) | | 76 (22.8%) | 45 (21%) | | 55 (22.6%) |
| None | | 76 (71%) ^*^ | 190 (58.6%) | 49 (55.7%) | | 217 (63.1%) | 62 (64.6%) | | 204 (61.1%) | 130 (60.7%) | | 152 (62.6%) |
| Pathology | |  |  |  | |  |  | |  |  | |  |
| High-grade | | 12 (17.4%) | 29 (14.5%) | 11 (23.4%) | | 30 (13.5%) | 8 (16%) | | 32 (14.7%) | 20 (15.5%) | | 20 (13.1%) |
| Middle-grade | | 32 (46.4%) | 93 (46.5%) | 17 (36.2%) | | 108 (48.6%) | 22 (44%) | | 103 (47.2%) | 66 (51.2%) | | 66 (43.1%) |
| Low-grade | | 25 (36.2%) | 78 (39%) | 19 (40.4%) | | 84 (37.8%) | 20 (40%) | | 83 (38.1%) | 43 (33.3%) | | 67 (43.8%) |

Note: DM diabetes mellitus; PC pancreatic cancer; BMI body mass index; Wt weight; ∆Wt loss of weight. * *p*<0.05, ** *p*<0.01, *** *p*<0.001

Suppl. Table 4: The logistic regression analysis related to radical surgery

| Independent factors | OR (95% CI) | *p* |
| --- | --- | --- |
| Long-term DM | 0.46 (0.22-0.99) | 0.05 |
| Heavy Wt loss (∆Wt≥5kg) | 0.39 (0.18-0.78) | 0.009 |
| Diameter of tumor | 0.7 (0.56-0.88) | 0.002 |
| Remote metastasis | 0.15 (0.06-0.39) | 0.001 |

Note: adjusting for age, gender, BMI, smoking, drinking, family history of DM, family history of PC, anti-diabetic drugs, anti-hypertension drugs, position of the tumor, CA 19-9, diameter of the tumor and remote metastasis. DM: diabetes mellitus; PC: pancreatic cancer; BMI: body mass index; ΔWt: loss of weight.
